# Supplementary material for: MCM2–7-dependent cohesin loading during S phase promotes sister-chromatid cohesion
Source: eLife. 2018 Apr 3;7:e33920. doi: 10.7554/eLife.33920 (PMC5897099; doi:10.7554/eLife.33920)
Supplement: Supplementary file 1. [file elife-33920-supp1.docx]

**Supplementary File 1.** siRNAs used in this study

| **siRNA** | **Sequence** |
| --- | --- |
| siLuciferase | 5’-UCAUUCCGGAUACUGCGAU-3’ |
| siMCM2 | 5’-GAAGAUCUUUGCCAGCAUU-3’; 5’-GGAUAAGGCUCGUCAGAUC-3’  5’-GCCGUGGGCUCCUGUAUGA-3’; 5’-GGAUGUGAGUCAUGCGGAU-3’ |
| siMCM3 | 5’-GGACAUCAAUAUUCUUCUA-3’; 5’-GCAGGUAUGACCAGUAUAA-3’  5’-GGAAAUGCCUCAAGUACAC-3’; 5’-GACCAUAGAGCGACGUUAU-3’ |
| siMCM5 | 5’-GAAGAUCCCUGGCAUCAUC-3’; 5’-GAACAGGGUUACCAUCAUG-3’  5’-GGACAACAUUGACUUCAUG-3’; 5’-CCAAGGAGGUAGCUGAUGA-3’ |
| siNIPBL/SCC2 | 5’-CUGAUAAACUAGAACGAAA-3’ |
| siMAU2/SCC4 | 5’-GAGAAGGCGUGGUUGAUAU-3’ |
| siSTAG2 | 5’-CCACUGAUGUCUUACCGAA-3’ |
| siRAD21/SCC1 | 5’-GGAAGAAGCAUUUGCAUUG-3’ |
| siSororin | 5’-CAGAAAGCCCAUCGUCUUA-3’ |
| siWAPL | 5’-CGGACTACCCTTAGCACAA-3’ |
| siCDC7 | 5’-CAGGAAAGGUGUUCACAAA-3’; 5’-CUACACAAAUGCACAAAUU-3’  5’-GUACGGGAAUAUAUGCUUA-3’; 5’-GCAUUCAUCAGUUUGGUAU-3’ |
| siDBF4 | 5’-GAACACACAUUAAGUGAAA-3’; 5’-GCACAAACCUUGGGUCGAA-3’  5’-GAGCAGAAUUUCCUGUAUA-3’; 5’-CCAAACAGAUGGCGAUAAG-3’ |
| siDRF1 | 5’-GGAAACAUCGGCCAUGGUU-3’; 5’-AAACAUCGGCCAUGGUUGA-3’  5’-GGAAACCCGUUGACUCGGU-3’; 5’-GAGCGAACCGGGAAAGGGA-3’ |
| siWDHD1 | 5’-GGUAAUACGUGGACUCCUA-3’; 5’-GCUGUGAAUUUAGCCAUUA-3’ |
| siTIMELESS | 5’-GGAAGACGCUGUUGGUAAA-3’ |
| siTIPIN | 5’-UGGAUUAAACGAAGACAUU-3’ |
| siDDX11 | 5’-GCAGAGCUGUACCGGGUUU-3’; 5’-CGGCAGAACCUUUGUGUAA-3’  5’-GAGGAAGAACACAUAACUA-3’; 5’-UGUUCAAGGUGCAGCGAUA-3’ |
| siCDC45 | 5’-GCACACGGAUCUCCUUUGA-3’; 5’-GCAAACACCUGCUCAAGUC-3’  5’-GGACGUGGAUGCUCUGUGU-3’; 5’-UCAAUGUCGUCAAUGUAUA-3’ |
| siGINS1 | 5’-GAAAUGGAGUGGUUUAAUA-3’; 5’-GAUGAAAGCUUUGUAUGAA-3’  5’-GCACUUCAGUCCUAUUAAA-3’; 5’-CAACGAGGAUGGACUCAGA-3’ |
| siRPA2 | 5’-GAUCAAUGCACACAUGGUA-3’; 5’-CAAAAUAGAUGACAUGACA-3’  5’-GAGUGAAGCAGGGAACUUU-3’; 5’-GUGGAACAGUGGAUUCGAA-3’ |
| siCHAF1A | 5’-GAAAGGAGCAGGACAGUUG-3’; 5’-ACACGAAGCUCCUGGACUA-3’  5’-AAACAACUGUCAUGUGGGU-3’; 5’-GACAUAGACUUUAGACCGA-3’ |
| siLIG1 | 5’-GGCAUGAUCCUGAAGCAGA-3’ |
| siFEN1 | 5’-UCACUAAGCAGCACAAUGA-3’; 5’-AGAAUGACAUCAAGAGCUA-3’  5’-GGGCAUCCCUUAUCUUGAU-3’; 5’-CCCAAGGGAUCCACUAAGA-3’ |
